# Feature selection methods and predictive models in CT lung cancer radiomics

**DOI:** 10.1002/acm2.13869

**Published:** 2022-12-17

**Authors:** Gary Ge, Jie Zhang

**Affiliations:** ^1^ Department of Radiology University of Kentucky Lexington Kentucky USA

**Keywords:** CT, feature selection, lung cancer, predictive model, radiomics

## Abstract

Radiomics is a technique that extracts quantitative features from medical images using data‐characterization algorithms. Radiomic features can be used to identify tissue characteristics and radiologic phenotyping that is not observable by clinicians. A typical workflow for a radiomics study includes cohort selection, radiomic feature extraction, feature and predictive model selection, and model training and validation. While there has been increasing attention given to radiomic feature extraction, standardization, and reproducibility, currently, there is a lack of rigorous evaluation of feature selection methods and predictive models. Herein, we review the published radiomics investigations in CT lung cancer and provide an overview of the commonly used radiomic feature selection methods and predictive models. We also compare limitations of various methods in clinical applications and present sources of uncertainty associated with those methods. This review is expected to help raise awareness of the impact of radiomic feature and model selection methods on the integrity of radiomics studies.

## 1 INTRODUCTION

Radiomics is a technique that extracts quantitative features, termed as radiomic features, from medical images using data‐characterization algorithms.^1^ Radiomic features can be used to identify tissue characteristics and radiologic phenotyping that is not observable by clinicians. Although morphological image features have been used in clinical practice for many decades, the concept of radiomics was first proposed in 2012. Since then, radiomics studies have experienced an exponential growth.

**FIGURE 1:**
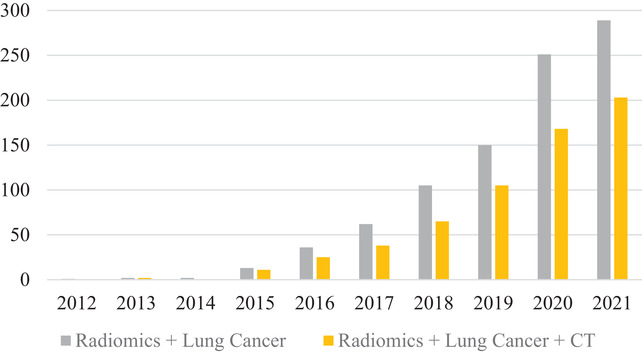
Number of publications by year from 2012 to 2021. The search was from PubMed for “Radiomics” + “Lung Cancer” and “Radiomics” + “Lung Cancer” + “CT.” The growth of the field of radiomics has been steadily increasing over the past decade. (Accessed on January 10, 2022)

We performed a literature search on publications from 2012 to the end of 2021 from PubMed. The use of the keyword “radiomics” returned 5579 results, adding “lung cancer” returned 911 results, then adding “CT” returned 560 results. Figure 1 illustrates the number of publications annually for the literature search.

We screened the publications to exclude 45 PET/CT or MRI‐related papers, 22 review papers, and 53 publications that were not in English or were not directly relevant to a radiomics study (e.g., new software tests, updated databases, or new proposed standards), leaving a total of 340 publications. Of the 340 publications, 161 had accessible full‐texts and were categorized into three categories: classification, prognostics, and meta‐analysis studies. Studies that primarily investigate the effect that image acquisition parameters, feature selection methods, and/or model selection have on predictive performance are considered meta‐analysis studies. Accordingly, 32 meta‐analysis studies were identified and excluded from the review, leaving 77 classification studies and 52 prognostics studies, which were included in the review.

The 22 review papers mainly covered three topics: image quality,^2^, ^3^ feature reproducibility,^4^ and potential applications in lung cancer.^5^, ^6^, ^7^, ^8^, ^9^, ^10^, ^11^, ^12^, ^13^, ^14^, ^15^, ^16^, ^17^, ^18^, ^19^, ^20^, ^21^, ^22^ The review papers regarding image quality or feature reproducibility discussed neither feature selection methods nor predictive models. Review papers focusing on potential clinical applications generally mentioned basic model construction when discussing a study. Two of these review papers included a short summary of feature selection methods and predictive models based on a very limited number of sources.^7^, ^11^ To the best of our knowledge, there is no comprehensive review that examines feature selection methods and predictive models used in CT lung cancer radiomics. Feature selection methods and predictive models play a pivotal role in radiomics. Hence, we provide a review regarding this topic based on a thorough analysis of existing literature.

## 2 OVERVIEW

**FIGURE 2:**
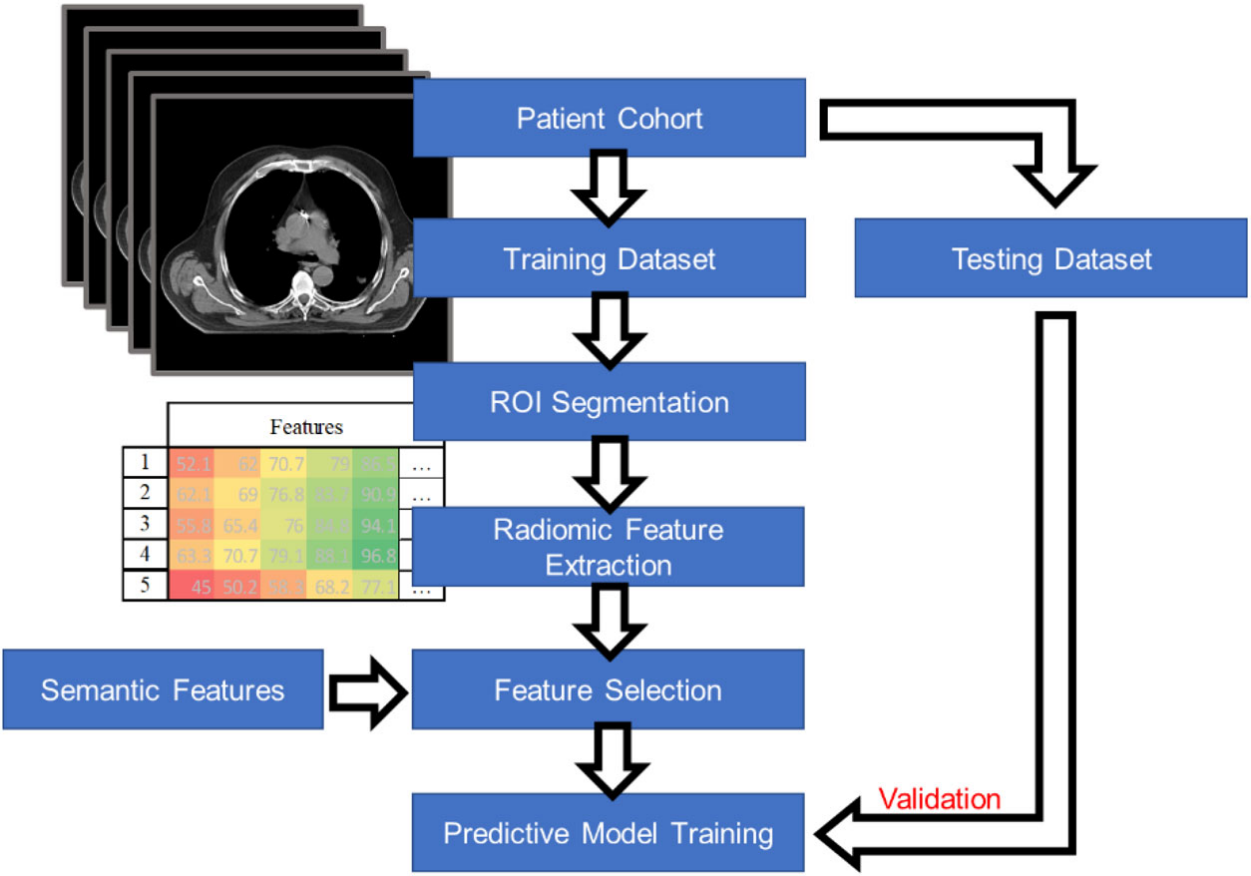
A typical pipeline of a radiomic study

Figure 2 shows a typical radiomics study pipeline. To begin, a patient cohort is chosen based on criteria appropriate to the study parameters (e.g., type of disease, treatment method, etc.). A patient cohort can be sourced from institutional data, public databases (e.g., The Cancer Imaging Archive), or a combination of both. Radiomic features are extracted from the cohort image sets using feature extraction software, often hundreds if not thousands of features are extracted. A feature selection process is then conducted to remove redundant and unstable features before the predictive model is ready to be trained. The datasets used to train a predictive model include a training and validation dataset, both originating from the patient cohort, and a test dataset, originating from an independent data source. The independent test set is key to model development as it provides an unbiased evaluation of model performance.

**FIGURE 3:**
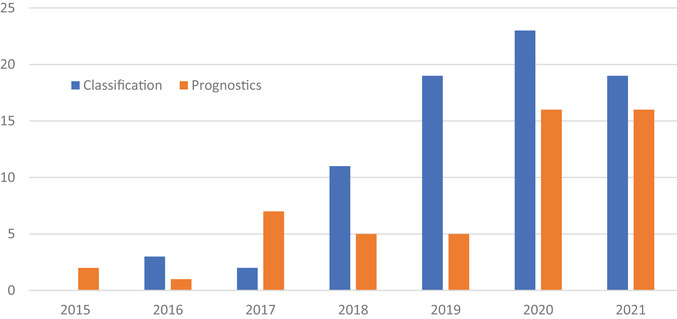
Annual number of publications for classification and prognostic studies in CT lung cancer radiomics that were included in this review

The two main clinical applications of radiomics are classification and prognostics. Classification studies create a model that can stratify a cohort into distinct bins determined by the study. Typically, these studies are interested in determining malignancy, specific gene expression or phenotyping, or segmentation.^23^, ^24^, ^25^, ^26^, ^27^, ^28^, ^29^, ^30^, ^31^, ^32^, ^33^, ^34^, ^35^, ^36^, ^37^, ^38^, ^39^, ^40^, ^41^, ^42^, ^43^, ^44^, ^45^, ^46^, ^47^, ^48^, ^49^, ^50^, ^51^, ^52^, ^53^, ^54^, ^55^, ^56^, ^57^, ^58^, ^59^, ^60^, ^61^, ^62^, ^63^, ^64^, ^65^, ^66^, ^67^, ^68^, ^69^, ^70^, ^71^, ^72^, ^73^, ^74^, ^75^, ^76^, ^77^, ^78^, ^79^, ^80^, ^81^, ^82^, ^83^, ^84^, ^85^, ^86^, ^87^, ^88^, ^89^, ^90^, ^91^, ^92^, ^93^, ^94^, ^95^, ^96^, ^97^, ^98^, ^99^ Prognostics studies create models that score individual cases along a sliding scale. The outputs of prognostic studies are heavily associated with future clinical outcomes such as treatment response, disease spread, or survival rates.^100^, ^101^, ^102^, ^103^, ^104^, ^105^, ^106^, ^107^, ^108^, ^109^, ^110^, ^111^, ^112^, ^113^, ^114^, ^115^, ^116^, ^117^, ^118^, ^119^, ^120^, ^121^, ^122^, ^123^, ^124^, ^125^, ^126^, ^127^, ^128^, ^129^, ^130^, ^131^, ^132^, ^133^, ^134^, ^135^, ^136^, ^137^, ^138^, ^139^, ^140^, ^141^, ^142^, ^143^, ^144^, ^145^, ^146^, ^147^, ^148^, ^149^, ^150^, ^151^ Figure 3 illustrates the annual quantity of publications for each application in this review.

It should be noted that we provide an overview of feature selection methods and predictive models and possible trends over time. This review does not intend to compare those methods nor to provide any recommendations for future study construction.

## 3 RADIOMIC FEATURE SELECTION

### 3.1 General features

Radiomic features are extracted using mathematical calculations to generate quantitative information within a region of interest (ROI). These features are extracted with open‐source software (PyRadiomics, IBEX, etc.), commercial software (ITK SNAP, GE Analysis Kit), or with in‐house software. Table 1 lists the software used by studies in this review.

**TABLE 1:** A list of feature extraction software used by the studies in this review

Feature extraction software	Study count
Inhouse Developed Software	54
PyRadiomics	32
Artificial Intelligence Kit (GE Healthcare)	8
Analysis Kit (GE Healthcare)	6
LIFEx	6
3D Slicer Radiomics	4
Definiens Developer	3
MaZda	3
Slicer‐Radiomics	3
Radiomics (Siemens)	2
AVIEW Research	1
CERR	1
IBEX	1
ITK	1
MATLAB Radiomics	1
PORTS	1
Radiomic Precision Metrics	1
RadiomiX Discovery Toolbox	1

The extracted radiomic features typically fall under four main categories: shape, first‐, second‐, and higher‐order features. Shape features indicate morphological characteristics of the ROI. First‐order features are direct measurements of the voxel values, describing the distribution of intensities within the ROI. Second‐order features, also referred to as texture features, provide descriptions of how voxels within a given ROI relate to each other. Higher‐order features can be generated by applying filters (e.g., wavelet) to the ROI/image before extracting features.

Each category of feature aside from higher‐order features contains a number of feature families, as seen in Table 2. Each of these feature families contain individual features that operate on the ROI in a similar manner. The number of commonly used individual features is over 100 and some of these exhibit spatial variations. There may be thousands of extracted features when all variations are considered.

**TABLE 2:** Commonly used radiomic feature families arranged by feature type

Feature type	Feature family
Shape	Morphology
First‐order	Local Intensity
	Intensity‐based Statistics
	Intensity Histogram
Second‐order	Grey Level Co‐occurrence Matrix (GLCM)
	Grey Level Run Length Matrix (GLRLM)
	Grey Level Size Zone Matrix (GLSZM)
	Grey Level Distance Zone Matrix (GLDZM)
	Neighborhood Grey Tone Difference Matrix (NGTDM)
	Neighboring Grey Level Dependence Matrix (NGLDM)
	Laws
	Gabor
Higher‐order	Wavelets
	Laplacian of Gaussian (LoG)

### 3.2 Feature selection methods

The most common feature selection methods are listed in Table 3 along with a short explanation of how each one works. Single or multiple feature selection methods are employed in radiomic studies. An alternative approach is to directly extract a smaller, predetermined list of features. Feature selection methods select the most reliable and relevant features for model training through removing redundant information. This process will improve the robustness of the predictive model and help reduce the risk of overfitting as well as calculation time.^152^

**TABLE 3:** Commonly used feature selection methods by the studies included in this review

Feature selection method	Mechanism
Component Analysis	Variance via sorted eigenvalues
Clustering	Choosing representative features among correlated groups
ICC/CCC	Measures feature reproducibility with correlation
LASSO	Regression analysis with L1 regularization
mRMR	Maximize F‐statistic and minimize correlation with defined feature limit
(Non)Parametric Statistics	Analysis of variance or means between two or more datasets
Pearson/Spearman Correlation	Determines highly correlated features prior to feature selection
Rank Sum	Two‐sided median analysis
Regression	Statistical relationship between dependent and independent variables
Relief	Scoring based on the nearest neighbor feature value differences
Random Forest	Calculate importance according to pureness of leaves

Abbreviations: CCC, concordance correlation coefficient; ICC, intraclass correlation coefficient; LASSO, least absolute shrinkage and selection operator.

Table 4 shows the commonly used feature selection methods according to three categories: single, serial, and parallel feature selection methods. The “Single Method” category is comprised of studies that only use a single feature selection method. The “Serial Method” and “Parallel Method” categories refer to studies that use multiple feature selection methods, but with different approaches. “Serial Method” studies apply feature selection methods in sequence, each step reducing the dimensionality of the radiomic features further. “Parallel Method” studies simply test multiple “single method” independently to achieve better results. A feature selection method that was used only twice or less is categorized as “Other.”

The least absolute shrinkage and selection operator (LASSO) method is most widely used in the “Single Method” and “Serial Method” categories. It is a popular choice likely due to the strong covariate reduction capabilities. The intraclass correlation (ICC) and concordance correlation coefficient (CCC) are also widely used, most often as a first step in a “Serial Method” study. ICC and CCC assess the reproducibility of features and serve as a filtering step before additional feature selection methods are employed. In regards to the “Parallel Method” category, as many as 13 feature selection methods were employed in a single study.^59^ In many cases, the feature selection methods chosen in “Parallel Method” studies were not widely used, resulting in the high frequency of “Other” feature selection methods in these studies.

**TABLE 4:** Overview of feature selection methods used in CT lung cancer radiomic studies

		Classification	Prognostics	Total
Single method	LASSO	8	3	11
	Other^a^	3	1	4
	Random Forest	3	1	4
	Logistic Regression	2	2	4
	Spearman/Pearson	2	3	4
	Component Analysis	2	1	3
Serial methods	ICC/CCC	28	16	44
	LASSO	28	13	41
	Pearson/Spearman	13	7	20
	mRMR	12	3	15
	(Non)Parametric Stats	12	0	12
	Clustering	7	0	7
	ICC/CCC + Pearson/Spearman	4	3	7
Parallel methods	Other^a^	8	13	21
	RELIEF	3	4	7
	(Non‐)Parametric Stats	2	3	5
	Pearson/Spearman	0	4	4
	mRMR	1	3	4
	Fisher Score	1	2	3
	Wilcox Rank Sum	1	2	3

The breakdown of the most common feature selection methods in “Single Method,” “Serial Method,” and “Parallel Method” studies is displayed. In many cases, the feature selection methods chosen in “Parallel Method” studies were not widely used, resulting in the high frequency of “Other” feature selection methods in these studies.

^a^ ^“^Other” indicates feature selection methods that were used twice or less.

#### 3.2.1 Feature selection in classification

**FIGURE 4:**
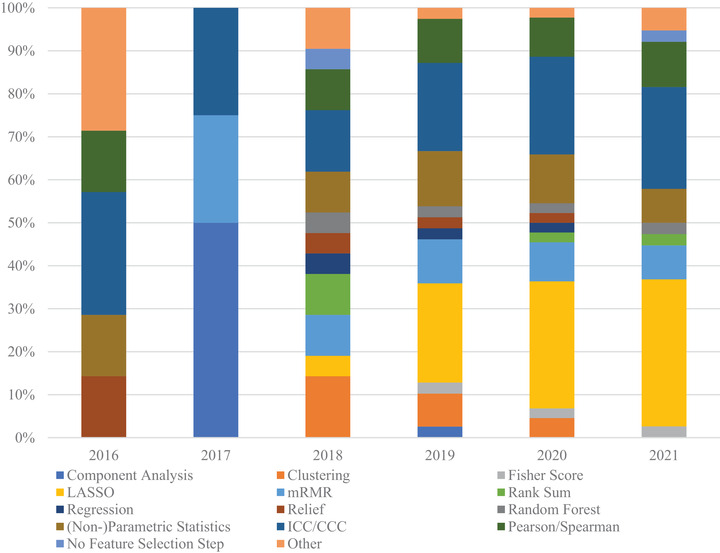
Feature selection method distribution by year for classification studies. The methods most often used were LASSO and ICC/CCC. Notably, LASSO and mRMR techniques that only appeared in 2018 onwards, with LASSO being chosen most often at the time of this review. The “Other” category encompasses feature selection methods that were used twice or less in the review

The feature selection methods used in classification studies were tallied and separated according to the year of publication. This helps to identify potential trends that could suggest the efficacy of a particular technique. Figure 4 shows that the feature selection method used most often in classification studies is LASSO. LASSO's first usage in CT lung cancer radiomic studies was in 2018 and became the most commonly used method in 2019 onwards. Maximum relevance–minimum redundancy (mRMR) was first used in 2017, though it was initially proposed in 2005.^153^ The mRMR feature selection method is most often used in a serial manner, that is, using mRMR then using LASSO on the mRMR results. Among those commonly used feature selection methods in the classification studies, LASSO and ICC/CCC have clearly gained more attention over the past several years.

#### 3.2.2 Feature selection methods in prognostics

**FIGURE 5:**
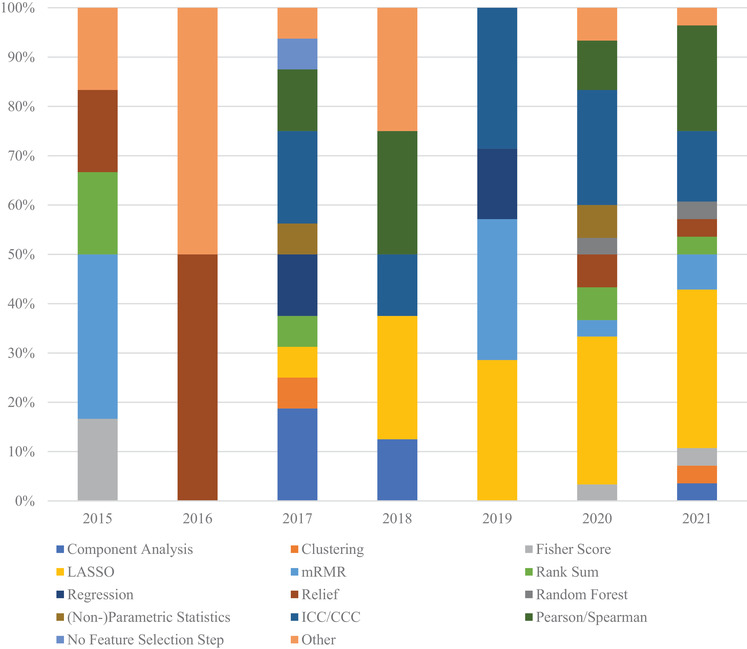
Feature selection method distribution by year for prognostic studies. The methods most often used were LASSO and correlation. The “Other” category encompasses feature selection methods that were used two or less times in the review

Figure 5 shows the distribution of feature selection methods in prognostic studies, which is very similar to that of classification studies. This suggests that the approach to both classification and prognostics studies is quite similar at the feature selection stage. The feature selection method that has become most commonly used in both classification and prognostic studies is LASSO.

### 3.3 Common features for lung cancer

While there are hundreds of commonly extracted radiomic features and many more niche features, the majority are removed during feature selection. Table 5A lists three filters, wavelet, LoG, and original, that are most commonly applied to feature families to generate high‐order radiomic features, based on features selected by the feature selection methods in the reviewed studies. Among the five most represented feature families listed for each filter, GLCM, first‐order, and GLSZM appeared the most often, contributing over half of the total count in each category. Table 5B lists individual radiomic features and their respective number of appearances for each of the three most commonly used feature families: GLCM, first‐order, and GLSZM. A total count indicates the overall use of the feature family among selected features.

**TABLE 5:** (A) Three filters, wavelet, LoG, and original, that are most commonly applied to feature families to generate high‐order radiomic features, based on features selected by the feature selection methods in the reviewed studies. A total count indicates the overall use of the filter among selected features. (B) Individual radiomic features and their respective number of appearances for each of the three most commonly used feature families: GLCM, first‐order, and GLSZM. A total count indicates the overall use of the feature family among selected features

A)	Classification		Prognostics	
Wavelet	glcm	65	glcm	36
	first‐order	50	first‐order	18
	glszm	37	glszm	8
	gldm	32	glrlm	8
	stats	22	gldm	3
	**Total Count**	**237**	**Total Count**	**111**
LoG	glcm	26	first‐order	18
	glszm	23	glcm	15
	first‐order	15	glszm	4
	gldm	12	gldm	4
	glrlm	9	glrlm	1
	**Total Count**	**104**	**Total Count**	**52**
Original	first‐order	11	glcm	11
	glcm	9	shape	7
	glszm	5	first‐order	6
	shape	5	glrlm	5
	glrlm	3	gldm	4
	**Total Count**	**35**	**Total Count**	**51**

B)	Classification		Prognostics	
GLCM	clustershade	16	clustershade	15
	clusterprominence	14	clusterprominence	13
	entropy	14	correlation	7
	correlation	10	inversevariance	7
	imc1	10	correl1	5
	**Total Count**	**173**	**Total Count**	**119**
First‐order	mean	12	skewness	9
	median	11	energy	6
	skewness	11	10percentile	5
	90percentile	6	maximum	5
	robustmeanabsolutedeviation	6	rms	5
	**Total Count**	**83**	**Total Count**	**59**
GLSZM	largearealowgraylevelemphasis	8	largeareaemphasis	5
	graylevelnonuniformity	6	graylevelnonuniformitynormalized	4
	zoneentropy	6	graylevelvariance	3
	lowintensitysmallareaemphasis	5	largearealowgraylevelemphasis	3
	sizezonenonuniformitynormalized	5	sizezonenonuniformity	3
	**Total Count**	**76**	**Total Count**	**39**

According to reviewed studies, there are not many common features in CT lung radiomic studies. In classification studies, there are nine features (mean, median, skewness, cluster shade, cluster prominence, entropy, correlation, imc1, imc2) that were selected more than 10 times in 79 total studies. In prognostic studies, there are three features (cluster shade, cluster prominence, skewness) selected more than 10 times in 51 total studies. The wide range of applications within classification and prognostics may diminish the appearance of common features.

### 3.4 Limitations

#### 3.4.1 Feature extraction consistency

There are many different options for feature extraction, such as self‐developed software, open‐source software, and commercially available software. The multitude of options limits the reliability of feature values, as feature calculations can be difficult to verify across all available implementations. Prior works investigating open‐source and in‐house software have shown that systemic differences in feature values can be observed across platforms, due in part to default settings and calculation differences.^154^, ^155^ It is important that features are generated in an identical manner throughout all radiomic studies in order to obtain results that are reproducible across testing. The largest standardization effort to date, the Image Biomarker Standardization Initiative (IBSI),^156^ has proposed a standard list of feature calculations and pre‐processing techniques. The adoption by individual research groups and open‐source software (e.g., PyRadiomics) has brought some consistency to how feature values are generated. Care should be taken as standardization efforts continue in order to minimize impacts on studies that utilize older versions of the standards.

The number of features that a given study extracts also varies heavily among the reviewed publications. The feature pool can range as low as 14 radiomic features^127^ to over 1000 radiomic features.^84^, ^103^, ^137^, ^138^ The wide range of radiomic features may suggest the inherent redundancy of many extracted features within a given study. Currently, radiomics is still at the research stage so it is necessary to test a large variety of features, even they are likely redundant, for different potential applications. Table 6 shows the statistics for the number of features selected for predictive model training across the reviewed studies. The average number of features is <10, though some studies have selected as many as 50 and one study found that semantic features alone performed better in predictive model training.^48^ Two studies were excluded from this analysis as they both utilized a very large number of radiomic features to train predictive models and these outliers would skew the datapoints. The outliers were a classification study that selected 115 features^74^ and a prognostics study that selected 149 features.^136^

**TABLE 6:** Statistics of the number of features extracted and features selected in reviewed radiomic studies

	Classification		Prognostics		Combined	
	Extracted	Selected	Extracted	Selected	Extracted	Selected
Mean	646.0	9.7	566.1	8.6	614.7	9.3
Std	625.3	9.3	543.7	6.2	595.9	8.4
Median	396	6	440	7	396	6
Range	24–2969	0–50	14–2317	1–28	14–2969	0–50

Typically, a radiomics study selects less than 10 features despite the fact that many studies extract hundreds of radiomic features before feature selection.

#### 3.4.2 Limited feature agreement

As seen in Table 5B, there is a limited agreement in features in both classification or prognostic studies, with cluster shade, cluster prominence, and skewness being the features with the highest occurrence out of hundreds of individual features. The wide variety of applications in radiomics may contribute to the limited number of common features found in this review, though additional work should be conducted to determine if the common features have application‐related dependencies or if there are no strong associations.

#### 3.4.3 Correlation cutoff values

ICC/CCC and Pearson/Spearman correlation are heavily used in radiomic studies, often to provide an initial filtering of radiomic features based on reproducibility and redundancy, respectively. Even though this review finds that these methods have a relatively widespread use, it is unclear as to why one, both, or neither method is chosen in any given study. The methods address two major concerns about radiomic feature values.^157^, ^158^, ^159^, ^160^, ^161^ but are not as ubiquitous as one might expect from useful techniques. An additional concern is the lack of consistency regarding the thresholds that studies utilize when implementing these correlation thresholds. ICC/CCC has ranged from <0.7^24^, ^26^ to <0.9^39^, ^42^ and even <0.95.^101^ Pearson/Spearman correlation threshold values have also ranged from >0.7^111^ to >0.9–0.95.^69^, ^124^ The variance in thresholds is large across all reviewed studies. This suggests that the threshold values are chosen based on existing studies or through a process of testing that results in better model performance. As radiomics moves towards clinical implementation, it may become a necessity to use consistent correlation thresholds and as such may be worth investigating in future work.

#### 3.4.4 Feature harmonization

Harmonization of radiomic feature values is a technique that is not consistently used in radiomic studies. The performance of feature selection methods and predictive models is directly affected when feature values are adjusted.^162^ It is also important to consider that the effects of harmonization are likely feature‐dependent, since features produce values that range across multiple orders of magnitude. Future efforts should be made to fully investigate the need and benefits of this practice.

## 4 PREDICTIVE MODELS

### 4.1 General predictive models

Typically, predictive models employ semantic data (e.g., malignancy status, smoking status, age, survival time, etc.), radiomic features, or a combination of the two. Many studies use multiple models with different combinations of semantic and radiomic data. After the data are input into the model for training, the machine learning process optimizes the parameters to produce the best fit to the data.

### 4.2 Commonly used predictive models in CT lung cancer radiomics

Table 7 provides an overview of the predictive models used in the reviewed studies. The studies are separated based on the number of models tested, either single or multiple models. When looking at the entirety of the reviewed studies, regression is the most commonly used predictive model in CT lung cancer radiomic studies, among which logistic regression comprises a vast majority. Other commonly used models after regression are support vector machine (SVM), random forest (RF), and Cox's proportional hazard model. Very often a study will use multiple modeling methods to test which method will produce better results. This process has been made relatively easy by available software, which can provide multiple options for modeling without much increase in effort.

**TABLE 7:** Overview of the predictive models in radiomic studies

		Classification	Prognostics	Total
Single model	Regression	40	11	51
	Cox	0	16	16
	Random Forest	9	3	12
	Support Vector Machine	8	2	10
	Statistics	3	3	6
Multiple models	Support Vector Machine	8	11	19
	Regression	6	8	14
	Naïve Bayes	6	5	11
	Discriminant Analysis	7	2	9
	*k*‐Nearest Neighbor	4	4	8
	Random Forest	8	0	8
	Decision Tree	1	5	6
	Neural Network	3	3	6
	Boosting	0	5	5
	Cox	0	5	5

The studies were separated by single model and multiple models. Regression, primarily logistic regression, was most commonly used in the reviewed studies.

**FIGURE 6:**
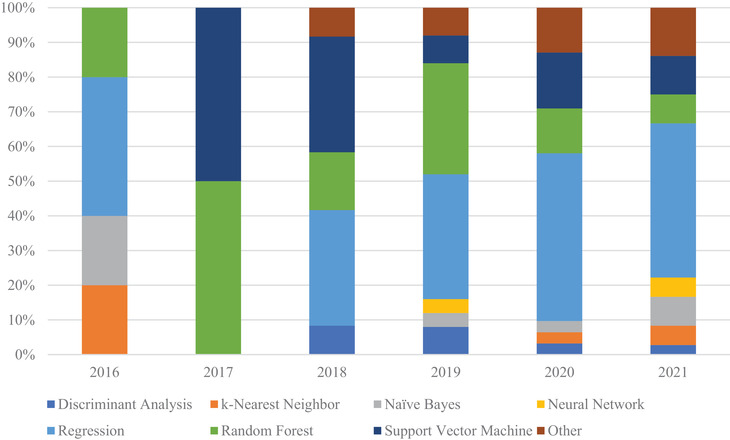
Distribution of models used in CT lung cancer radiomic studies for classification studies published during the review periods. The classification models utilize random forest and support vector machines more often than prognostic studies, as RF and SVM are better suited for classification tasks

When looking at classification studies, classifiers are unsurprisingly the model of choice since the goal of the predictive model is to classify inputs into distinct groups. Figure 6 shows the distribution of models used in CT lung cancer radiomic studies published during the review periods. The top three commonly used models for classification over time are regression, SVM, and RF. It shows that there is an increasing trend in the use of regression.

**FIGURE 7:**
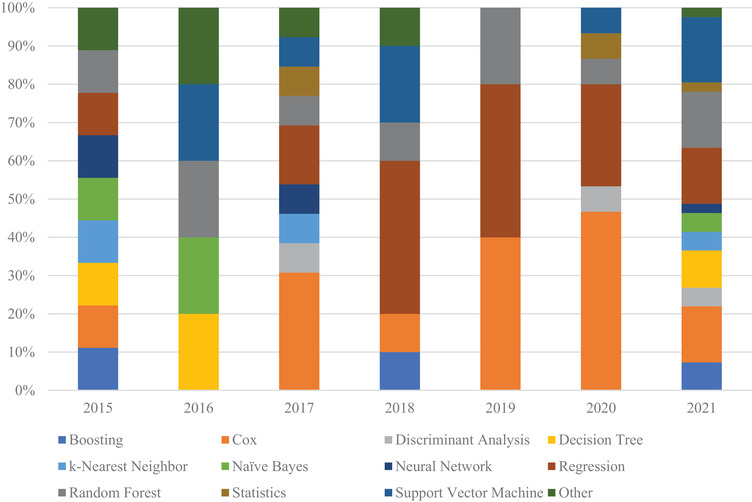
Distribution of models used in CT lung cancer radiomic studies for prognostics studies published during the review periods. Regression, RF, and SVM are still widely used like in classification studies. Cox's proportional hazard models are more often used in prognostic studies, as it is suited for survival rate stratification

When looking at prognostic studies, as seen in Figure 7, studies still favor regression and RF as models. The primary difference from classification studies is the use of the Cox's proportional hazard model, since it is suited for variable outputs. This shows the general trend that logistic regression has in parsing large patient cohorts.

### 4.3 Limitations

#### 4.3.1 Cohort power

For radiomic studies, datasets can be sourced from public datasets (e.g., TCIA, RIDER, NLST, public clinical trials) or from institutional data collected for the purpose of the study. The decision to conduct a study using institutional data, public data, or a combination of both depends on the goals of the study and the method used to validate the predictive model. Table 8A shows the usage of institutional and public data in the reviewed studies. The number of datapoints available in a chosen cohort plays an important role in determining the statistical power of a study. Table 8B characterizes the cohort sizes in this review, illustrating how diverse cohort sizes are in radiomic studies. The size of the cohort should be appropriate to the task, and relevant power metrics should be conducted to ensure that the cohort is not too small or too large. This condition may be difficult to fulfill, depending on the availability of relevant clinical data. The lack of statistical methods used for power analysis is of a concern.

**TABLE 8:** Radiomic study cohort characteristics among reviewed studies

		Classification	Prognostics	Combined
(A)	Institutional only	64	41	105
	Public only	6	8	14
	Combined	7	3	10
(B)	Cohort size			
	Mean	256.3	242.8	250.9
	Std	206.7	217.1	211.1
	Median	200	161	188
	Range	7–1212	24–1171	7–1212
(C)	No split	30	23	53
	Cohort split	47	29	76
	Independent testing	5	6	11
(D)	Cohort split ratio			
	90/10	1	1	2
	86/14	0	1	1
	80/20	6	5	11
	75/25	3	2	5
	70/30	24	3	27
	67/33	1	4	5
	65/35	1	1	2
	63/37	2	1	3
	60/40	3	3	6
	55/45	2	3	5
	50/50	4	5	9

#### 4.3.2 Training and validation

Once patient cohorts are determined and radiomic features are selected, predictive model training is the next step. Some studies generate a weighted radiomic signature prior to incorporating semantic features, which condenses the selected radiomic features into a single term. In these situations, concerns of double‐training should be addressed, as the selected radiomic features are trained on the dataset to determine weighting prior to model training then used again as a radiomic signature during model training. Future considerations should be made to determine if a weighted radiomic signature has adverse effects on model performance compared to the direct use of the selected features.

Once model training is complete, best practice is to perform model validation followed by independent testing using an external dataset to assess model performance outside the training data. Some radiomic studies forgo validation and testing entirely, instead simply presenting the training results in a standalone fashion. This methodology lacks both validation and independent testing and results should be treated with some skepticism. Other studies will perform a validation step using a training and validation dataset originating from the patient cohort, with varying ratios between the two datasets. This is often done as a multiple‐fold cross‐validation that can be used to estimate the fit of the model on new data, but no technique can replace testing with independent datasets. Table 8C characterizes how studies approach validation and testing and shows that 11 of 129 reviewed studies employ independent testing. Table 8D shows that there is no consistency in how patient cohorts are split, though the effects of split ratio on model performance in radiomics are unclear.

Once a model is trained, the performance of the model is reported as results. Currently, there is no standard reporting method in radiomic studies for results when multiple feature selection methods and/or multiple models are used in a study. Some studies opt to only report the best‐performing combination while others display the results from all combinations used. The latter reporting style should be considered as standard in future works as a complete view of how specific methods interact and perform will help future studies choose more optimal techniques.

## 5 CONCLUSION

Currently, radiomics and deep learning are the most researched techniques in medical imaging. The hand‐crafted radiomic analysis necessitates the use of machine learning or statistical algorithms after feature extraction in order to construct a predictive model. According to our review of existing publications, their performance highly depends on the feature selection methods and prediction models used for the analysis. While efforts have been focused on the standardization of imaging biomarkers by addressing radiomic feature reproducibility and stability, the evaluation and validation of feature selection methods and predictive modeling to strengthen the stability and reproducibility of radiomic features remain a necessity.

The future refinement of radiomics need to investigate those limitations discussed above, including but not limited to feature extraction consistency, feature value harmonization, cohort power, and training/validation best practices. With the improvement of its robustness, radiomic analysis can become an efficient tool for aiding clinicians in risk stratification, prognostics, and patient management.

## AUTHOR CONTRIBUTIONS

All listed authors contributed to the literature search and to drafting the manuscript.

## CONFLICT OF INTEREST

The authors declare that there is no conflict of interest.
